# F-18 fluorodeoxyglucose uptake and water-perfusable tissue fraction in assessment of myocardial viability

**DOI:** 10.1007/s12149-012-0631-2

**Published:** 2012-07-15

**Authors:** Hidehiro Iida, Ulla Ruotsalainen, Maija Mäki, Merja Haaparnata, Jörgen Bergman, Liisa-Maria Voipio-Pulkki, Pirjo Nuutila, Kazuhiro Koshino, Juhani Knuuti

**Affiliations:** 1 Turku PET Center, Turku University Central Hospital, 20520 Turku, Finland; 2 Department of Investigative Radiology, National Cerebral and Cardiovascular Center Research Institute, 5-7-1 Fujishiro-dai, Suita, Osaka 565-8565 Japan; 3 Department of Nuclear Medicine, Turku University Central Hospital, 20520 Turku, Finland; 4 Departments of Medicine, Helsinki University Central Hospital, 00029 Helsinki, Finland; 5 Department of Signal processing, Tampere University of Technology, 33720 Tampere, Finland

**Keywords:** Myocardial viability, Chronic myocardial infarction, ^18^F-FDG, Water perfusable tissue fraction, Positron emission tomography

## Abstract

**Objectives:**

^15^O-water-perfusable tissue fraction (PTF) has been shown to be a potential index for assessing myocardial viability in PET, an alternative to ^18^F-fluorodeoxyglucose (FDG). This study aimed to directly compare these two independent methods in assessing myocardial viability in patients with abnormal wall motion.

**Methods:**

PET study was performed on 16 patients with previous myocardial infarction, before coronary artery bypass graft operation (CABG). The protocol included a ^15^O-carbonmonoxide static, a ^15^O-water dynamic and an ^18^F-FDG dynamic scan, during the euglycemic hyperinsulinemic clamp. Echocardiography was performed at the time of PET and 5–12 months after the CABG, and the wall motion recovery was evaluated on segmental and global bases. Consistency between PTF and ^18^F-FDG was evaluated visually and also in a quantitative manner. Predictive values for the wall motion recovery were also compared between the two approaches.

**Results:**

The image quality of ^18^F-FDG was superior to that of ^15^O-water. The qualitative PTF showed significantly smaller defects than ^18^F-FDG, and the quantitative PTF showed slightly greater values than ^18^F-FDG in the infarcted region. The two methods were, however, consistent visually and also quantitatively. The predictive values of the wall motion recovery were almost equal between the two approaches. The absolute ^18^F-FDG uptake was varied in normal segments, and predictive values for the wall motion recovery by the absolute ^18^F-FDG was less (accuracy: 80 %) compared with those by the relative ^18^F-FDG (accuracy: 87 %) and the quantitative PTF (accuracy: 89 %).

**Conclusion:**

Despite the small sample size, PTF appears to give consistent results with the ^18^F-FDG approach, and might be an alternative viability assessment.

## Introduction

Successful coronary reperfusion by thrombolysis, angioplasty, or bypass graft surgery is often associated with improvement of myocardial contractile function in patients with coronary disease, suggesting the presence of dysfunctional but viable myocardium within the area of abnormal wall motion [1–3]. Several techniques have been proposed for detecting the reversibly injured myocardium in the clinical setting including single photon emission computed tomography (SPECT) with ^201^Tl or ^99m^Tc-MIBI, echocardiography during dobutamine stimulation, and flow-metabolism imaging with positron emission tomography (PET). Of these, ^18^F-FDG and PET [4–7] have been considered to be the gold standard. Commonly, increased ^18^F-FDG uptake relative to blood flow has been judged as a sign of preserved myocardial viability. The advantages of ^18^F-FDG PET include the good quality of the images and high accumulation of the tracer into dysfunctional but viable myocardium. Image quality can further be improved by performing the studies during euglycemic hyperinsulinemic conditions [8]. It is suggested that the maximized ^18^F-FDG uptake makes the tracer distribution proportional to the amount of viable myocardium.

^18^F-FDG is on the other hand a marker of glucose uptake, which depends on a number of physiologic factors such as myocardial work load, metabolic conditions of the subject and hormones, suggesting limitations of utilizing ^18^F-FDG PET for the viability assessment. Indeed, it has been demonstrated that the normalized uptake of ^18^F-FDG relative to a control region is more accurate in predicting wall motion recovery after coronary artery bypass graft surgery [9].

An alternative approach has been proposed, employing ^15^O-labeled radiotracers [10–13]. This enables the direct measurement of the proportion of ^15^O-water perfusable tissue that is capable of exchanging water rapidly. The water perfusable tissue fraction (PTF, g/ml) was defined as the fraction of the water-perfusable tissue within a given volume of region-of-interest (ROI), and is measured from the kinetic analysis on the ^15^O-carbonmonoxide and ^15^O-water data sets. Similarly, the perfusable tissue index (PTI) was defined as the proportion of the ^15^O-water perfusable tissue within the total anatomical tissue that was measured from the transmission (tissue density) scan. These parametric values were originally assessed for given ROIs. A qualitative, myocardial distribution of radioactive water at a later phase reasonably corresponded to a relative PTF distribution [13], which was also shown to be consistent with the relative distribution of FDG accumulation in an experimental pig model of old myocardial infarction [14]. More recently, sophisticated computer programs have provided functional parametric images of quantitative PTF and PTI from a single dynamic PET image obtained following intravenous ^15^O-water [15–17].

There can be tradeoffs between the ^18^F-FDG and PTF (and PTI) approaches, in terms of the total study duration, radiation dose, cost of the procedure, image quality, sensitivity to various error sources, etc. There have, however, been no direct comparisons between ^18^F-FDG and PTF in the assessment of myocardial viability in a clinical patient population. Against this background, we performed a head-to-head comparison between ^18^F-FDG uptake and PTF in patients with chronic coronary artery disease who underwent revascularization. Consistency between ^18^F-FDG and PTF studies was evaluated both visually and in a quantitative manner. Predictive value of wall motion recovery after successful revascularization therapy was also compared between the two measures.

## Materials and methods

### Subjects

The study group consisted of 16 patients (15 males and 1 female). In 10 of them, a myocardial infarction was diagnosed previously both by electrocardiographic and enzymatic criteria. In the other 6 patients, chronic left ventricular dysfunction and an occluded major coronary artery were detected but there were no confirmed data available on previous myocardial infarction. The patient characteristics are summarized in Table 1. All patients had stable, angiographically confirmed coronary artery disease and a permanent wall motion abnormality at rest. Three had diabetes mellitus, and three had heart failure. The interval between the acute event and the PET study was more than 5 months. All of them underwent coronary artery bypass grafting (CABG) after the PET imaging. There was no particular change in the patient’s symptoms between the time of the PET study and the time of CABG. The study was approved by the Ethics Committee of the Turku University Central Hospital, Turku City, Finland [8/1993 § 153 (dated 19.10.1993)]. All studies were performed in accordance with the ethical standards laid down in the Declaration of Helsinki and subsequent guidelines at Turku University. Informed consent was given by each patient prior to his inclusion in the study. No subjects have any particular issues that might reveal their identity.

**Table 1:** Patient characteristics

Patient #	Age	Weight (kg)	Duration of CAD (months)	NYHA	Heart failure	Medication	Time between angiogram and PET (weeks)	EF (%)	EF (%)	HR	Systolic blood pressure (mmHg)	Diastolic blood pressure (mmHg)	Blood glucose (mmol/l)
								Pre ope.	Post ope.				
1	41	85	5	2	N	Beta blocker, nitrate	8	25	19	67	105	67	5.33
2	56	87	216	2	N	Beta blocker, nitrate	12	24	19	71	120		5.48
3	64	80	17	3	Y		6	33	35	64	133	85	5.48
4	44	75	9	2	N	Beta blocker, nitrate	4	28	38	75	104	67	4.52
5	64	90	96	3	Y	Nitrate	8	49	55	55	136	83	4.41
6	44	105	9	2	N		12	56	31	65	140	90	5.24
7	65	98	72	3	N	Beta blocker, nitrate	8	48	61	59	146	85	4.91
8	55	89	6	2	N	Beta blocker, nitrate	12	30	57	62	130	78	4.84
9	61	81	5	2	N	Ca-antagonist, nitrate	16	54	42	90	150	80	4.52
10	69	67	69	2	N	Ca-antagonist, nitrate	16	69	60	54	112		4.98
11	52	82	24	3	Y	Nitrate	8	48	58	50	135		5.01
12	56	83	240	3	N	Beta blocker, nitrate	4	31	52	51	119		5
13	57	89	66	3	N	Beta blocker, nitrate	4	60	53	61	132		5.4
14	70	82	21	3	N	Ca-antagonist, nitrate	16	51	64	64	171		5.1
15	48	77	7	2	N	Beta blocker, nitrate	8	57	68	52	133		6.39
16	71	76	360	3	N	Ca-antagonist, nitrate	4	68	68	65	178		6.59
Mean ± SD	57 ± 10	84 ± 9	76.4 ± 105.	2.5 ± 0.5			9.1 ± 4.4	46 ± 15	49 ± 16	62.8 ± 10.2	134 ± 21	79 ± 8	5.2 ± 0.6

### Study design

**Fig. 1:**
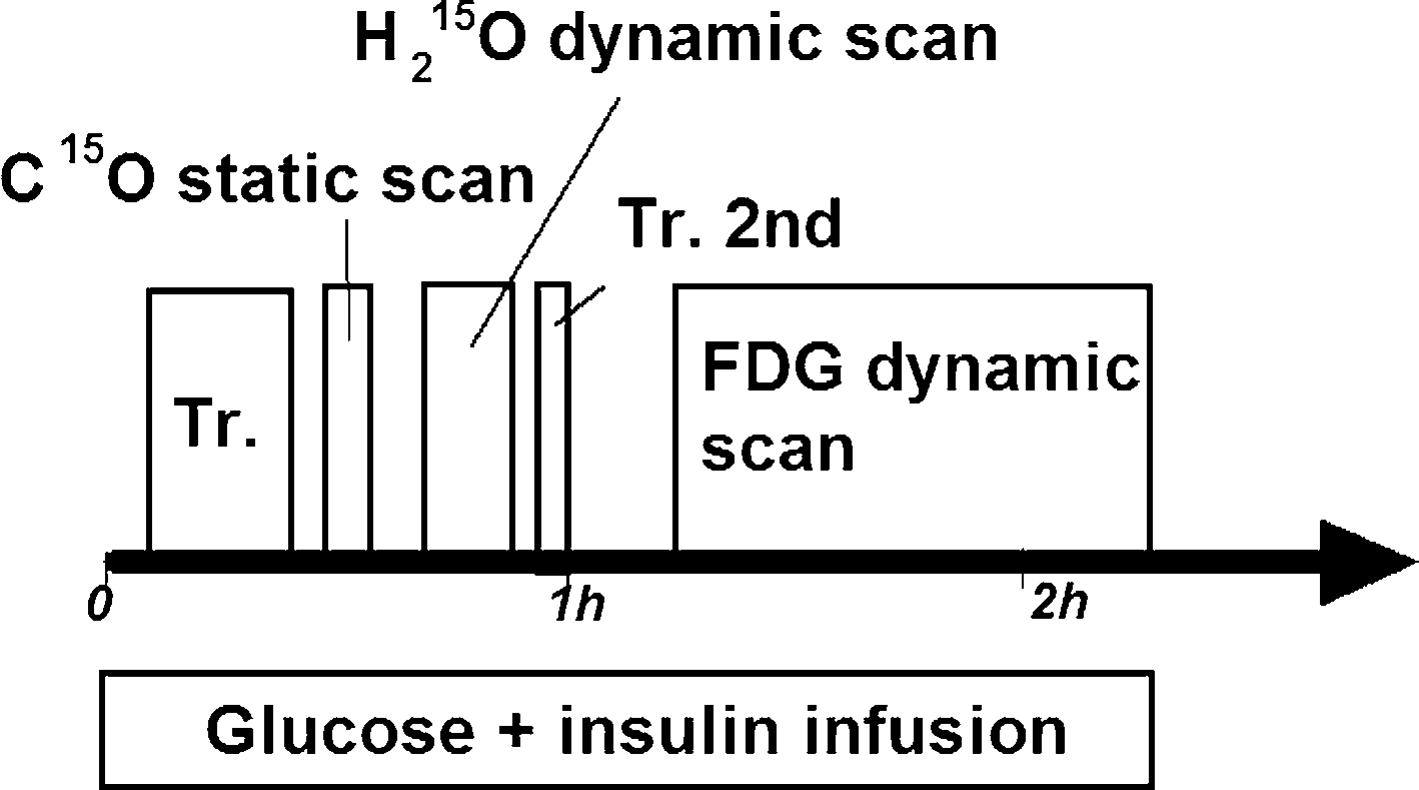
Schematic diagram of the study protocol. *Tr* indicates the transmission scan. The second transmission scan was used to confirm that the patient did not move during the period

All patients underwent coronary angiography, radionuclide ventriculography, ^201^Tl SPECT, echocardiography, and PET imaging before the CABG. The PET imaging included scans with ^15^O-carbonmonoxide, ^15^O-water and ^18^F-FDG during euglycemic hyperinsulinemic clamp (see Fig. 1). Angiography was performed 9.1 ± 4.4 weeks before the PET study (all within 4 months). Echocardiography was performed on the same day of the PET imaging and repeated 5–12 months after CABG. Wall motion recovery was then evaluated for each dysfunctional segment in the preoperative study, and this was compared with the results from the PET and SPECT imaging. The consistency of different PET images was also evaluated.

### PET studies

PET scans were performed in 2D using an ECAT 931-08/12 scanner (CTI/Siemens Inc., Knoxville, Tenn.) [18]. This scanner enables 15 planes of data to be acquired in an axial field-of-view (FOV) of 10.5 cm. All emission and transmission sinograms were reconstructed with a Hanning filter with a cutoff frequency of 0.3. This resulted in a spatial resolution of 8.4 ± 0.7 mm full width at half maximum (FWHM) for the emission data and 7.7 ± 0.7 mm FWHM for the transmission data at the center of the FOV with the slice thickness of 6 mm.

All subjects fasted for at least 10 h before the PET scanning. Medications were continued as noted in Table 1. Patients lay supine on the scanner bed with their arms out of the FOV. Two catheters were placed, one in the antecubital vein for infusion of saline or glucose and insulin and ^15^O-water and another in the radial vein of the contralateral hand that was warmed with air temperature of 70 °C for sampling of arterialized venous blood. During the whole study period, insulin was infused continuously as previously described to maintain the euglycemic hyperinsulinemic condition [8, 19, 20]. The rate of insulin infusion was 1 mU/kg/min. During hyperinsulinemia, euglycemia was maintained by infusing 20 % glucose. The rate of the glucose infusion was adjusted according to plasma glucose concentrations measured every 5–10 min from arterialized venous blood. Blood samples were taken at 30-min intervals for determination of insulin, free-fatty acid, and lactate concentrations.

A 20-min transmission scan was performed by exposure of the external ring source of ^68^Ge. These data were used to correct for subsequent emission scans for photon absorption in the body, and to estimate the density distribution. After the transmission scan, the blood pool was imaged by inhalation of ^15^O-labeled carbon monoxide. ^15^O-carbon monoxide of approximately 3.7 GBq was administered for 2 min, and a 4 min, single frame emission acquisition was initiated at 2 min after the end of ^15^O-carbonmonoxide inhalation. Venous blood samples were taken every 2 min during the scan, and the ^15^O-carbonmonoxide concentration in whole blood was measured using a NaI well counter cross calibrated with the scanner.

After a 15-min period to allow for decay of ^15^O-radioactivity to background levels, ^15^O-water was infused and a dynamic PET scan was started using a previously reported protocol [21, 22]. Briefly, ^15^O-water in 5-ml saline was infused into the antecubital vein at a constant rate for a period of 2 min. Total administration dose was 1.5 GBq. A 20 frame dynamic PET scan was started at the initiation of the infusion, and lasted for 6 min. The scan sequence consisted of 6 × 5 s, 6 × 15 s, and 8 × 30 s.

Metabolic imaging was then performed using ^18^F-FDG. ^18^F-FDG (240 ± 40 MBq) was infused intravenously over 2 min, and dynamic scanning was initiated at the start of infusion. This scan lasted for 60 min.

### PET data analysis

All sinograms were corrected for tissue attenuation and reconstructed using a Filtered Back Projection method. Images were transferred to a Linux workstation, and further analyses were performed using the dedicated image analysis packages (Dr. View; Asahi-Kasei, Tokyo) and in-house software programs.

A blood volume image was calculated using the ^15^O-carbonmonoxide emission data, in which the original image was divided by the average of the blood counts measured by the well counter. Images of extravascular density were created by subtracting images of the blood volume from the corresponding transmission images after conversion of the latter to tissue density.

The ^15^O-water dynamic images were integrated over the period of ^15^O-water administration, and the blood volume was subtracted as previously described [11, 23, 24]. These images (the build-up or BU phase images) were considered to qualitatively illustrate the regional distribution of myocardial blood flow. The ^15^O-water dynamic images were also integrated over the washout period, and the blood volume was similarly subtracted. These images (the washout or WO phase images) were considered to correspond to the qualitative distribution of PTF [11, 13].

Quantitative values of MBF, PTF, and the arterial blood volume (*V*_a_) were calculated for each segment (see below) according to the non-linear least squares fitting as validated previously [21]. The arterial input function was estimated from the left-ventricular time-activity curve, in which the limited recovery of left-ventricular chamber activity and the spillover from myocardium were corrected as reported previously [22, 25]. The adoption of PTF in this model has implications for the interpretation of MBF values, such that the measurement exclusively represents the mean regional flow to that mass of the tissue within the ROI that is capable of exchanging water rapidly, i.e., that mass of tissue defined by PTF. This method provides regional blood flow only to the perfusable tissue, and has units of ml/min/g of perfusable tissue. In areas of myocardial infarction, this MBF represents the regional flow to the residual water-perfusable tissue. Another blood flow has also been calculated in this study as a product of MBF and PTF, which should represent the average blood flow to the volume of ROI (i.e., ml/min/ml of ROI). MBF_t_ is adopted to describe this parameter.

The absolute glucose consumption was calculated from the ^18^F-FDG uptake for each segment by the graphical analysis [8]. The relative glucose or ^18^F-FDG uptake, as well as the relative MBF images, was also calculated by normalizing the absolute quantitative values to the reference segment in each study. The reference segment was defined as the normal region (or closest to the normal) according to the findings of coronary angiography and echocardiography. The reference segment was the lateral wall in 14 studies, and anteroseptal segment in 2 studies.

### Regions-of-interest

The myocardium was divided into eight segments according to the previously described criteria [8], namely the anterior basal, anterior, anteroseptal, lateral, inferoseptal, apical, inferior, and posteroseptal regions. Of these, the last seven segments were included in this analysis. ROIs were drawn for each of the seven segments manually on transaxial tomographic images of ^18^F-FDG, and these ROIs were projected onto all other PET images.

### Visual analysis

Consistency of the defect between different images was evaluated for the segments described above. The defect severity was graded visually into 4 levels and its consistency was compared at each segment. Approximately the following limits are used in the visual classification: normal: 100–75 % of reference, mild: 75–50 %, severe: 50–25 % and complete: <25 % of activity in the reference segment. Consistency of the defect was evaluated between the approaches, namely the ^18^F-FDG image versus the ^15^O-water washout phase image (i.e., the qualitative PTF), the ^15^O-water build-up phase image (i.e., qualitative MBF) versus the ^18^F-FDG image, and ^15^O-water build-up phase image versus qualitative PTF image. The predictive values of the wall motion recovery after the revascularization were also compared among the approaches. In this analysis, the normal and mild defect segments were considered to be viable in all measures except for the qualitative PTF image, in which only the completely normal segment was assigned to be viable.

### Quantitative analysis

The glucose consumption values (absolute and relative) were plotted as a function of the absolute MBF (MBF_t_). The glucose consumption values were also compared with PTF.

### Coronary angiography

All patients underwent selective coronary angiography by standard techniques. The cine tapes were analyzed by an experienced radiologist. A 50 % or greater reduction in the diameter in a major epicardial branch was considered significant.

### Echocardiography

Two-dimensional echocardiography (Acuson 128XP/5, Acuson Inc or Aloka SSD 870, Aloka Inc) was performed on the same day as PET according to the semiquantitative method recommended by the American Society of Echocardiography Committee on Standards, but the segmental subdivision was modified to correspond to the PET studies [8]. Echocardiograms were analyzed by a blinded experienced physician. The results of individual prerevascularization and postrevascularization echocardiograms were ultimately verified by comparison of videotape recordings. Wall motion and thickening were scored according to the following scale: 1, normal; 2, hypokinetic wall motion with systolic thickening; 3, akinetic wall motion with no systolic thickening; and 4, dyskinetic wall motion and no systolic thickening.

After the revascularization, improvement of contractile function was diagnosed if systolic thickening (corresponding to a score of 1 or 2) became apparent in a segment that had been akinetic or dyskinetic or if normal motion was detected in a previous dysfunctional segment. Improvement in function was acknowledged only if it was apparent in a central area of the segment. Special attention was focused on the anteroseptal segments because postsurgical wall motion abnormalities are common in this area. Thus, appearance of postoperative anteroseptal hypokinesia was regarded as normal, and improvement was recognized only if systolic thickening became apparent in a previously akinetic or dyskinetic segment or if hypokinesia was normalized.

### Radionuclide ventriculography

A gated, blood-pool, radionuclide ventriculography was performed in two views. Six hundred cycles (10 min) were collected after injection of 740 MBq of [^99m^Tc]-labeled human serum albumin. The left anterior oblique view was used for ejection fraction calculations. A Siemens-Orbiter gamma camera (Siemens Gammasonics, IL, USA) was used, and ejection fractions were calculated with the Gamma-11 program (Nuclear Diagnosis, Stockholm, Sweden).

### Analytical procedures

Plasma glucose was determined in duplicate by the glucose oxidase method using an Analox GM7 (Analox Instruments LTD, London, England) glucose analyzer. Serum insulin was measured by radioimmunoassay kit (Pharmacia, Uppsala, Sweden).

### Statistical analysis

Independent variables were compared by analysis of variance. All results are expressed as the mean and one standard deviation (SD). Sensitivity, specificity, and positive and negative predictive values of functional recovery were calculated for the measures. To test different threshold values in quantitative MBF, quantitative PTF and quantitative ^18^F-FDG uptake in predicting functional recovery, the discriminant analysis of SAS statistical program was used (SAS Institute Inc., Cary, N.C.). Receiver operating characteristic (ROC) analysis was also applied for assessment of FDG uptake and PTF in differentiating functionally recovered from non-recovered segments.

## Results

Hemodynamic and other parameters are summarized in Table 1. Plasma glucose and insulin concentrations were 6.0 ± 1.0 mmol/l and 12 ± 3 mU/l in the fasting state. During clamp serum insulin concentrations increased to 79 ± 19 mU/l. The plasma glucose concentrations were 5.2 ± 0.6 mmol/l during the PET studies (Table 1).

In total, 105 of the 112 segments in the 16 patients were included in the final analysis. The remaining 7 segments, mostly inferior, were excluded because they were out of FOV in the PET study. In the preoperative echocardiography, 48 segments had normal wall motion and of the 57 dysfunctional segments, 35 were hypokinetic, 17 were akinetic and 5 were dyskinetic. In the revascularization, 54 of the 57 dysfunctional segments were successfully revascularized.

In the postoperative echocardiography, 81 segments were normal, 6 were hypokinetic, 13 akinetic and 5 were dyskinetic. Thus, 34 (60 %) of the initially dysfunctional revascularized segments recovered (partially in one) and 20 (35 %) segments did not show functional improvement. In the 3 non-revascularized segments, wall motion was unchanged in two and worsened in one.

The preoperative ejection fraction was 45.7 ± 15.1 % (range from 24 to 69 %). Postoperatively the ejection fraction was slightly higher (49 ± 16 %) but the difference was not significant. However, the average wall motion score in echocardiography was significantly improved by revascularization (preoperatively 0.8 ± 0.9 vs. postoperatively 0.45 ± 0.9, *p* = 0.001). Furthermore, the number of segments recovered per patient was significantly associated with increased global left ventricular ejection fraction (*r* = 0.65, *p* = 0.0065).

**Fig. 2:**
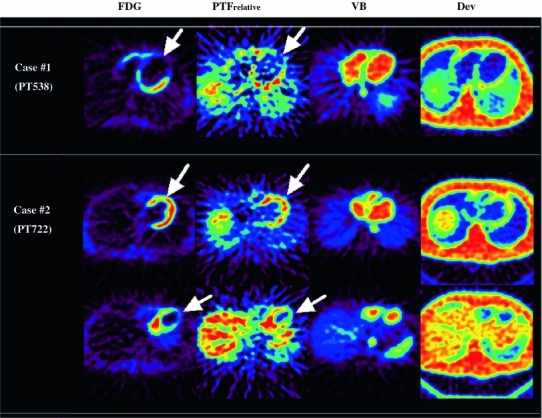
Example images of extra-vascular tissue density (*D*_ev_), the blood volume (*V*_B_), ^15^O-water washout phase (WO, or qualitative PTF), and ^18^F-FDG uptake, obtained from 2 typical cases. In case 1, the wall motion was irreversible in the anterior wall segment (*arrow*, **a**), which was well predicted by both WO and FDG as complete defect. In case 2, wall motion was reduced in the anterior wall (*arrow*, **b**), but was not improved in the apex (*arrow*, **c**). These were also consistent with the findings in both WO and FDG. Image quality of ^18^F-FDG was better than that of WO, but both images provided consistent results

Figure 2 shows examples of calculated images obtained from typical two studies, demonstrating the extravascular tissue density (*D*_ev_), blood volume (*V*_B_), ^15^O-water image at a washout phase (i.e., the qualitative PTF), and ^18^F-FDG uptake. In case 1, both PTF and FDG images showed a large defect in the anterior wall region, and these two images were considered to be consistent in all segments. No recovery of wall motion was detected after revascularization. In case 2, the anterior, anteroseptal and apical walls were akinetic. The FDG and PTF images indicated preserved myocardial viability in the former two segments but scar in the apex. After the revascularization, the anterior and anteroseptal segments were completely recovered but no change was detected in the apex.

Consistency of the defect scores defined by the visual analysis is summarized in Table 2. The qualitative MBF (^15^O-water build-up phase images) and the qualitative PTF (^15^O-water washout phase image) agreed in general with the ^18^F-FDG images. It should, however, be noted that the qualitative PTF image tended to show less severe defects, and resulted in milder defect scores in 14 segments compared with the ^18^F-FDG images. The ^15^O-water images at the build-up phase (qualitative MBF) showed considerably larger defects as compared with PTF and ^18^F-FDG.

**Table 2:** Image consistency as evaluated by the defect score by visual analysis

FDG uptake vs. qualitative PTF or ^15^O-water washout phase image						
FDG	PTF					
	0	1	2	3	Total	
0	73	1	2	0	76	
1	6	7	0	1	14	
2	0	4	1	0	5	
3	0	2	2	6	10	
Total	79	14	5	7	105	Concordance = 83 %

Qualitative MBF or ^15^O-water build-up phase vs. FDG uptake						
MBF	FDG					
	0	1	2	3	Total	
0	65	3	0	1	69	
1	2	4	0	0	6	
2	8	4	3	2	17	
3	1	3	2	7	13	
Total	76	14	5	10	105	Concordance = 75 %

Qualitative MBF or ^15^O-water build-up vs. qualitative PTF or ^15^O-water washout phase						
MBF	PTF					
	0	1	2	3	Total	
0	68	0	0	1	69	
1	5	1	0	0	6	
2	6	8	3	0	17	
3	0	5	2	6	13	
Total	79	14	5	7	105	Concordance = 74 %

The predictive values for the wall motion recovery resulted from the visual analysis are summarized in Table 3, in which the optimal defect score was determined so as to provide the best predictive accuracy for each parameter. The best sensitivity was obtained with ^18^F-FDG, but the specificity of ^18^F-FDG was slightly lower than that of qualitative PTF. The accuracy was almost equal between the ^18^F-FDG and qualitative PTF image analysis.

**Table 3:** Results of visual analysis in terms of evaluating wall motion recovery

Images	Sensitivity (%)	Specificity (%)	PPV (%)	NPV (%)	Accuracy (%)
FDG uptake	97	70	85	93	87
Qualitative MBF (^15^O-water build-up)	82	75	90	60	80
Qualitative PTF (^15^O-water washout)	88	85	91	81	87

Upper values correspond to only dysfunctional segments, and lower to total segments

Defects were frequently seen (12 of 15 cases) in the qualitative PTF images in the apical segment. Of these, 7 segments showed recovery, while the other 5 did not. All 3 segments assigned to be viable in the apex by the qualitative PTF image demonstrated recovery. In the ^18^F-FDG image, 7 segments were assigned to be scar (out of 15) in the apex, of which 1 segment showed recovery. All segments (8 segments) assigned as normal or viable in the apical segment by the ^18^F-FDG showed wall motion recovery.

**Fig. 3:**
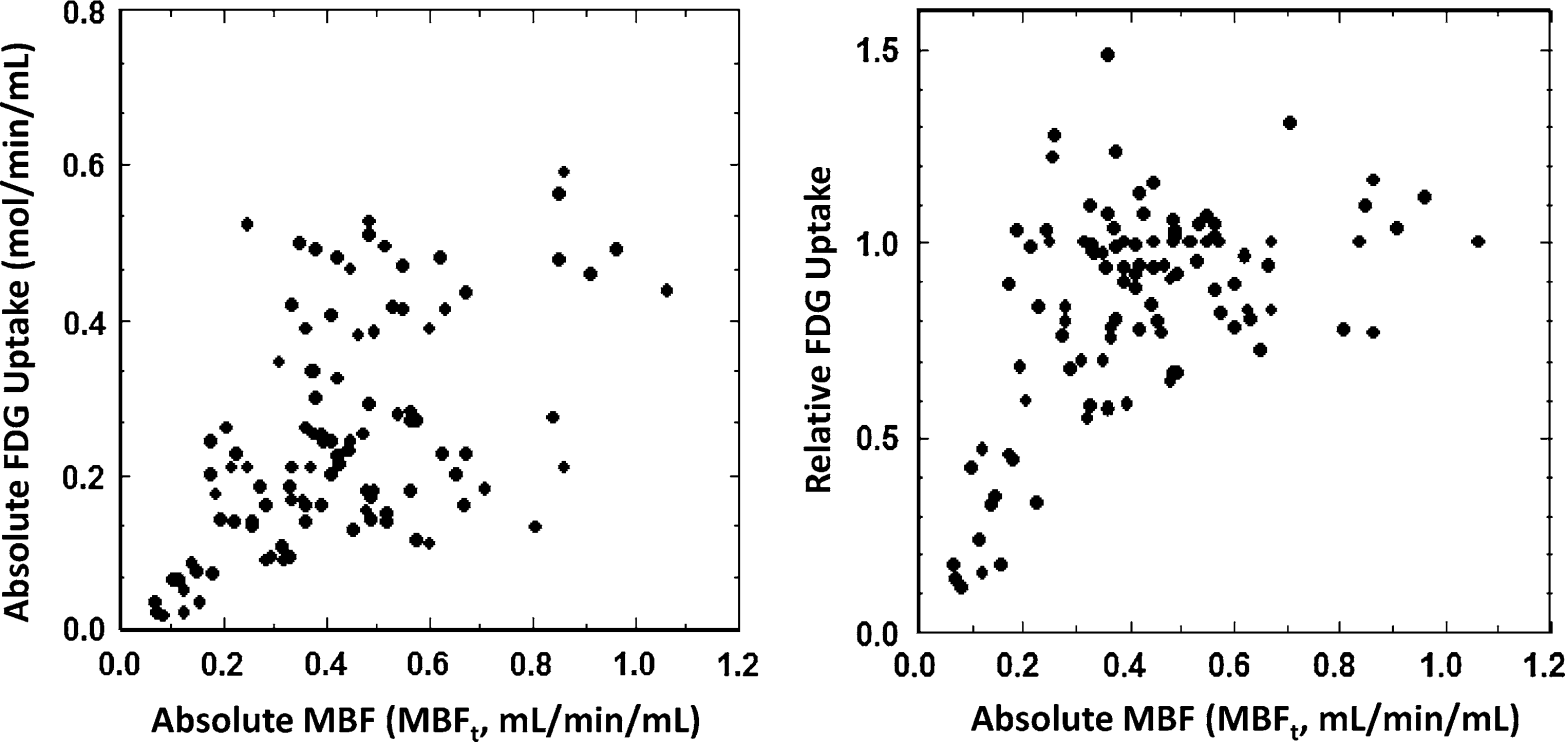
Relation between ^18^F-FDG uptake and absolute myocardial blood flow determined by means of ^15^O-water PET. *Left*: absolute uptake of ^18^F-FDG which was calculated using the arterial input function. *Right*: relative uptake of ^18^F-FDG to a control region. MBF_t_ denotes ml of blood per minutes per ml of regions-of-interest adopted to this plot

**Fig. 4:**
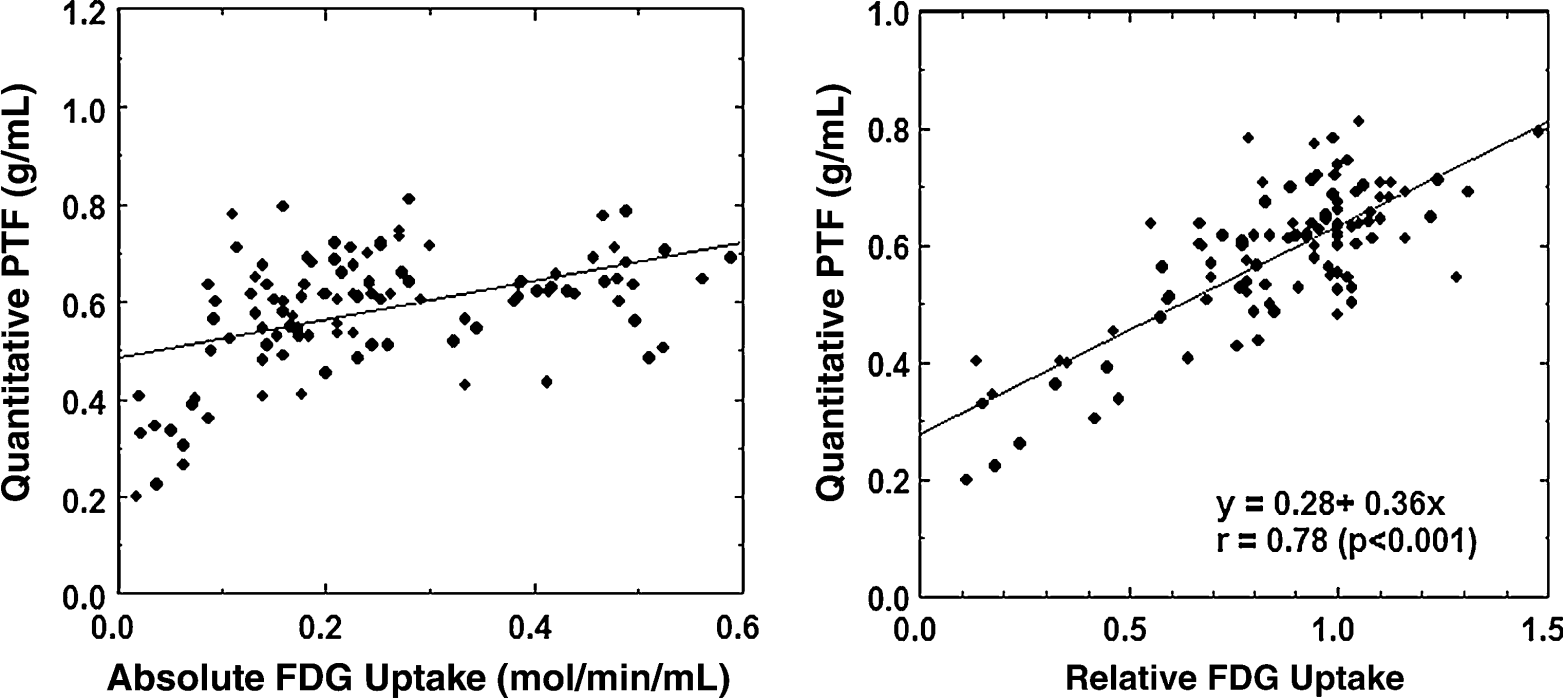
Relation between the water-perfusable tissue fraction (PTF, g/ml) versus ^18^F-FDG uptake (*Left*: absolute ^18^F-FDG uptake. *Right*: relative ^18^F-FDG uptake)

Figure 3 shows the absolute and relative glucose uptake as a function of the absolute MBF, in which the flow per volume (i.e., the transmural flow) was adopted to the myocardial flow (i.e., the MBF estimated from the ^15^O-water kinetic analysis was multiplied by PTF) [10, 11, 13]. The variability of absolute glucose uptake from patient to patient was larger as compared to relative values. Thus, the relation was diverged at the normal MBF range. On the other hand, the relative ^18^F-FDG uptake which was normalized in each subject to the control segment was rather constant at the range: MBF_t_ > 0.5 ml/min/ml. The relative ^18^F-FDG uptake showed a large variation at the range of MBF_t_ between 0.3 and 0.5 ml/min/ml, and was decreased linearly with the MBF_t_ for MBF_t_ < 0.3 ml/min/ml. There was no significant correlation between absolute glucose uptake and PTF, while a significant correlation was observed if the relative ^18^F-FDG uptake was compared with PTF (*p* < 0.001) (Fig. 4).

**Fig. 5:**
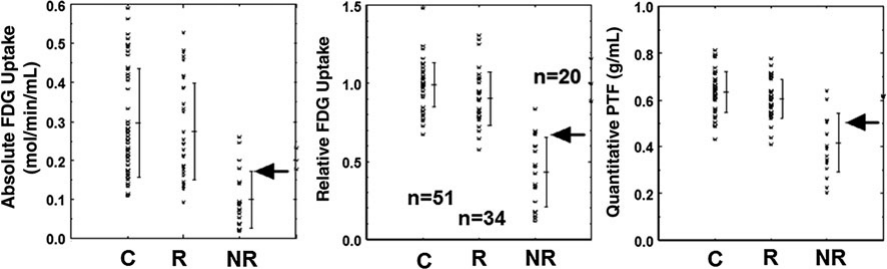
Absolute and relative ^18^F-FDG uptake and water-perfusable tissue fraction (PTF) in control segments (C) and in revascularized segments. *Arrows* correspond to the threshold values to discriminate between the recovered (R) and non-recovered (NR) (see text)

**Fig. 6:**
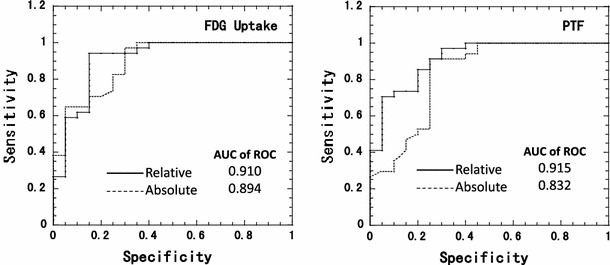
ROC analysis for wall motion recovery after successful coronary artery bypass graft surgery in patients with hypocontractile function

Predictive values for the wall motion recovery resulted from the quantitative analysis are summarized in Table 4 and Fig. 5. The most accurate results were obtained using the limit values of 0.62 ml/min/g in MBF, 0.34 ml/min/ml in MBF_t_, 0.51 g/ml in PTF, 34.4 mol/100 g/min in the absolute ^18^F-FDG uptake and 67 % in the relative ^18^F-FDG uptake. The positive predictive values obtained with the different variables were nearly equal among the variables. However, the absolute ^18^F-FDG uptake and MBF showed clearly lower negative predictive values than relative ^18^F-FDG uptake and PTF values. Figure 6 shows the results of ROC analysis for FDG uptake and PTF. The ROC analysis demonstrated greater area under the curve in relative rather than absolute values for both FDG and PTF. The area under the curve was slightly greater with PTF rather than relative FDG. Combination of FDG with MBF is of interest in terms of the improved accuracy in determining wall motion recovery. The best accuracy was obtained by the combination of relative ^18^F-FDG uptake and relative MBF (Table 4).

**Table 4:** Results of quantitative analysis in terms of evaluating wall motion recovery

Images	Sensitivity (%)	Specificity (%)	PPV (%)	NPV (%)	Accuracy (%)
^18^F-FDG uptake (relative)	94	75	86	88	87
^18^F-FDG uptake (absolute)	76	85	90	68	80
Quantitative MBF	68	90	92	62	76
Quantitative PTF	91	85	91	85	89
Quantitative MBF_t_	82	90	93	75	85
Glucose (relative) + MBF	97	85	92	94	93

Upper values correspond to only dysfunctional segments, and lower to total segments

There were 4 errors in PTF (out of 15) in the apical segment, in which PTF was below the threshold value (values ranged from 0.41 to 0.48 g/ml) and assigned not to be viable, although the wall motion was recovered or normal in the preoperative study. Similarly ^18^F-FDG showed error in 3 apical segments, in which the values were below the threshold level, although the wall motion was normal or reversible.

## Discussion

The present study demonstrated a direct comparison between the ^18^F-FDG and PTF approaches in the assessment of myocardial viability in a clinical patient population. Despite the small number of subjects (*n* = 16), it was shown that the relative uptake of ^18^F-FDG well correlated with the quantitative PTF. In the visual evaluation, although the image quality was superior with ^18^F-FDG than the ^15^O-water-based qualitative PTF images, the two images were consistent with each other. Furthermore, both images of relative ^18^F-FDG uptake and PTF (as well as the qualitative washout phase images for ^15^O-water) well predicted the wall motion recovery after successful revascularization in the dysfunctional segments. The area under the curve in the ROI analysis also demonstrated similar values between the relative FDG uptake and PTF. The accuracy appeared to be similar between the ^18^F-FDG and PTF measurements, both in the visual and quantitative analyses.

Use of ^18^F-FDG and PET is often considered the best approach for myocardial viability assessment. This is largely attributed to the clear contrast of the ^18^F-FDG accumulation in the myocardium, particularly during the insulin clamp condition, and further due to the high sensitivity of the PET device as compared with the conventional SPECT camera. The accurate image reconstruction of PET technique including the attenuation correction by means of the transmission scan and the significantly smaller contribution of scatter are regarded as other reasons.

It should, however, be noted that the absolute glucose consumption was variable in the normal segment as shown in Figs. 3 and 4. This suggests that the myocardial ^18^F-FDG uptake is not maximized even during the insulin clamp condition. Previous studies also demonstrated that the glucose uptake is influenced by several physiological factors [26], even during the hyperinsulinemic condition [27]. Thus, accuracy in the viability assessment appeared to be poorer with the absolute measurement of myocardial ^18^F-FDG uptake as compared with the relative uptake (see Figs. 5, 6; Table 4), as has already been reported by Knuuti et al. [28]. Use of relative uptake of ^18^F-FDG (or glucose) normalized to a control segment in each subject was therefore suggested for the assessment of myocardial viability. However, the normalization procedure requires a definition of a control segment, which is sometimes subjective and might become a critical procedure, particularly in patients with multiple vessel disease.

The present study showed that the accuracy to predict the functional outcome was improved (the best among the comparisons) when the ^18^F-FDG was combined with perfusion imaging (Table 4). This was probably attributable to the presence of sub-endocardial scars, which might have been assigned as viable because of increased accumulation of ^18^F-FDG in the residual tissue (due to the variation). The addition of MBF to PTF is of interest. However, due to the limited number of subjects, further systematic studies are needed with larger number of subjects.

### ^15^O-water perfusable-tissue fraction

^15^O-water is a diffusible and chemically inert tracer [29]. The early distribution after the administration of ^15^O-water reflects the regional blood flow [24, 30]. On the other hand, rapid clearance from high flow areas and further uptake into low flow areas cause the myocardial ^15^O-water distribution, after a sufficient long period, to be proportional to the tissue distribution which is perfused by water [10]. The PTF value estimated from the kinetic ^15^O-water PET data should represent quantitatively the density of tissue that is capable of rapidly exchanging water within the PET study period. It is hypothesized that the PTF corresponds to the amount of the reversibly injured myocardium in the region of hypocontractile function. This hypothesis is close to the one made for the ^18^F-FDG PET study, in which the glucose consumption is nearly maximized during the hyperinsulinemic euglycemic condition, so that ^18^F-FDG accumulation highlights only the myocardial tissue that is not infarcted. Consistency between the two approaches, found in this study, suggests the validity of these hypotheses for both techniques.

In the visual analysis, we observed that the size of the defect in the ^15^O-water washout phase (qualitative PTF) image was significantly less, and the defect score was smaller than that of ^18^F-FDG (Table 2). Therefore, the most accurate predictive values were obtained when only the completely normal segments were considered to be viable with the qualitative PTF image, while both normal and mild defects were assigned to be viable with ^18^F-FDG. Also the quantitative PTF values had significant offsets as compared with ^18^F-FDG in regions of myocardial infarction (Fig. 4, right). The reason for these is not clear, but is possibly due to the diffusion of ^15^O-water from the perfusable tissue into the scar regions, or reduced ^18^F-FDG uptake at the border region close to the scar tissue. Despite this difference, the predictive values for the wall motion recovery were almost equal in both the visual and quantitative analyses.

There are some practical advantages in the PTF and/or PTI approaches for the viability assessment as compared with ^18^F-FDG. Rapid kinetics of ^15^O-water allows a short scan, which has the logistical advantage of increasing patient throughput. The short half-life further makes the study repeatable within a short interval, and decreases the radiation dose. The total study duration may be within a half hour including the transmission, ^15^O-carbonmonoxide and ^15^O-water scans, if the protocol is optimized [25]. The advent of relatively inexpensive equipment dedicated to the production of ^15^O-labeled tracers should make this approach more acceptable to clinical centers. The major limitation of the ^15^O-water technique was considered as the poor statistics, which is largely due to the short scan period and short half life of ^15^O. This study, however, demonstrated that information derived from the ^15^O-water study is essentially consistent with that obtained from the ^18^F-FDG study. Further development of the image reconstruction technique is highly appreciated to improve the image quality, as has been successfully demonstrated by Katoh et al. [31]. The short half-life also requires the installation of a ^15^O-dedicated cyclotron in the same facility, as ^15^O-labeled tracers cannot be delivered from other centers unlike ^18^F-FDG. There is also concern that the ^15^O technique is probably sensitive to patient movement during the study, and thus a sophisticated algorithm needs to be developed to correct for any possible movement during the study.

PTF was originally introduced to compensate for the partial volume effect, as a parameter that represents the fractional mass of water-perfusable tissue within the selected ROI. The magnitudes hold the same also for the FDG uptake values, as well as the washout-phase, and the build-up phase images.

In theory, PTF can be affected by heterogeneous flow distribution surrounding the infarction, causing systematic underestimation in PTF [32], as has been demonstrated by Herrero and coworkers [33]. They reported that PTF could be underestimated by as much as 25 % [33], when the tissue contained 4 different flows with minimum flow level >0.10 ml/min/g. However, they also demonstrated in the same report that in the case of a mixture of extremely low flow tissue (e.g., MBF < 0.01 ml/min/g), which should correspond to scar tissue, the estimated PTF values well represented the fraction of only the high flow tissue. Thus, although further investigations will need to include a direct histological and/or histochemical comparison, PTF can still be a reasonable index to represent the fraction of non-scar tissue around the myocardial infarction. The work by Herrero suggested that the perfusion was low enough in the scar tissue to be neglected within the PET examination period.

### Limitations of this study

Significant defects were frequently seen in the apical segment in the ^15^O-water washout image (i.e., the qualitative PTF image). The reason for this may be patient movement during the PET studies, particularly between the ^15^O-carbonmonoxide and ^15^O-water scans. This artificial defect was less in the quantitative PTF values, in which values were estimated basically without the ^15^O-carbonmonoxides blood volume. There were still 4 segments that showed errors in quantitative PTF in the apical region (all below the threshold value) for the wall motion recovery. This was probably due to the smallest thickness and/or the largest wall motion in the apical segment. Note that both PTF and ^18^F-FDG can be underestimated in the segment in a thin structure and with the wall motion. An independent threshold value may have to be defined for both techniques in this segment.

### Future prospects

The ^15^O-water image at the build-up phase was used as the qualitative MBF image, and the ^15^O-water washout phase image as the qualitative PTF. This study demonstrated that the washout phase image alone well predicted the wall motion recovery, as accurate as quantitative PTF estimates and ^18^F-FDG. Recently proposed techniques that calculate the functional images of quantitative MBF and PTF [15, 17, 34] should also be evaluated. Adequacy of such technique including the application of the 3D acquisition should also be evaluated for different PET devices and image reconstruction programs.

No software alignment program was applied in this study, to correct for the possible movement of the patient between the scans. Further study is required to correct for the movement, as has been done in a recent study [35].

It should be noted that the quantitative PTF values and relative ^18^F-FDG uptake are dependent on the ROI size due to the partial volume effect. For instance, the use of wider ROIs causes a reduction in values. Therefore, the ROI size probably needs to be standardized for each segment. In addition, the reference values and threshold values for viability may need to be defined at each segment.

## Conclusion

This study demonstrates that although the image quality is superior with ^18^F-FDG than with ^15^O-water, the ^15^O-water perfusable tissue fraction (PTF) was consistent with the qualitative analysis of ^18^F-FDG uptake, in terms of the agreement in the defect identification and the assessment of myocardial viability. The image quality is superior with ^18^F-FDG than with ^15^O-water, but PTF provided enough information eventually to predict the wall motion recovery after successful revascularization in patients with old myocardial infarction.
